# Two-Dimensional PCA Highlights the Differentiated Antitumor and Antimicrobial Activity of Methanolic and Aqueous Extracts of *Laurus nobilis* L. from Different Origins

**DOI:** 10.1155/2014/520464

**Published:** 2014-04-16

**Authors:** Maria Inês Dias, João C. M. Barreira, Ricardo C. Calhelha, Maria-João R. P. Queiroz, M. Beatriz P. P. Oliveira, Marina Soković, Isabel C. F. R. Ferreira

**Affiliations:** ^1^Mountain Research Center (CIMO), ESA, Polytechnic Institute of Bragança, Campus de Santa Apolónia, 1172, 5301-855 Bragança, Portugal; ^2^REQUIMTE, Department of Chemical Sciences, Faculty of Pharmacy of University of Porto, Rua Jorge Viterbo Ferreira, 228, 4050-313 Porto, Portugal; ^3^Center of Chemistry, University of Minho, Campus de Gualtar, 4710-057 Braga, Portugal; ^4^Department of Plant Physiology, Institute for Biological Research “Siniša Stanković”, University of Belgrade, Bulevar Despota Stefana 142, 11000 Belgrade, Serbia

## Abstract

Natural matrices are important sources of new antitumor and antimicrobial compounds. Species such as *Laurus nobilis* L. (laurel) might be used for this purpose, considering its medicinal properties. Herein, *in vitro* activity against human tumor cell lines, bacteria, and fungi was evaluated in enriched phenolic extracts. Specifically, methanol and aqueous extracts of wild and cultivated samples of *L. nobilis* were compared considering different phenolic groups. Principal component analysis (PCA) was applied to understand how each extract acts differentially against specific bacteria, fungi, and selected human tumor cell lines. In general, the extract type induced the highest differences in bioactivity of laurel samples. However, from the PCA biplot, it became clear that wild laurel samples were higher inhibitors of tumor cell lines (HeLa, MCF7, NCI-H460, and HCT15). HepG2 had the same response to laurel from wild and cultivated origin. It was also observed that methanolic extracts tended to have higher antimicrobial activity, except against *A. niger, A. fumigatus*, and *P. verrucosum*. The differences in bioactivity might be related to the higher phenolic contents in methanolic extracts. These results allow selecting the extract type and/or origin with highest antibacterial, antifungal, and antitumor activity.

## 1. Introduction 

*Laurus nobilis* L. (Lauraceae), commonly known as laurel or bay leaves, is a native plant from the Southern Mediterranean region, found in warm climate regions with high rainfall [1]. It is one of the most widely used culinary spices for seasoning of meat products, soups, and fishes but is also used as an ornamental plant, especially in Europe and USA. It is also commercially grown in Turkey, Algeria, Morocco, Portugal, Spain, Italy, France, and Mexico [2–4]. The dry laurel and their infusions are traditionally used to treat gastrointestinal problems, such as epigastric, bloating, digestion, eructation, and flatulence problems. It also possesses anticonvulsive and antiepileptic activities and stimulant and narcotic properties [2, 5, 6]. The ability to suppress high blood sugar and prevent not only migraines and headaches but also bacterial and fungal infections has also been reported [3, 7].

Natural matrices, like* L. nobilis*, are rich sources of bioactive compounds. In fact, nearly 60% of the antitumor and anti-infectious drugs available on the market, or under clinical trial, are from natural origin [6, 8]. The biological activities of plant extracts are well recognized, namely, their antifungal, antimicrobial, insecticidal, and cytostatic effects. Accordingly, the bioactivity of plant extracts is often explored in a multifactorial manner [8, 9].

Nowadays, there is a worldwide concern about the use of synthetic chemical compounds as antitumor agents due to their potential negative health effects, opening ways to use plants as sources of natural compounds with similar activity [10]. On the other hand, the indiscriminate use of antibiotics to treat bacterial and fungal infections led to the emergence and spread of organisms resistant to broad-spectrum antibiotics, demanding new antimicrobial agents [11, 12].

There are some reports on the antitumor potential of* L. nobilis* essential oil [13, 14], methanolic [15], ethanol, and aqueous extracts [8]. However, most publications regard isolated compounds [6, 16, 17]. For instance, sesquiterpene lactones and methyl esters isolated from* L. nobilis *leaves exhibited moderate-to-significant cytotoxicity towards K562 leukemia cells [16]. Likewise, there are a considerable number of reports on the antimicrobial effects of* L. nobilis* essential oil [1, 4, 9, 18–21], aqueous [11], ethanolic [12, 22, 23], and methanolic extracts [24]. The antimicrobial activity of* L. nobilis* is mainly related to terpenes and phenolic compounds [7, 24–26].

Despite the previous findings, and as far as we know, this is the first study exploring* in vitro* antimicrobial and antitumor activities from cultivated and wild* L. nobilis* enriched phenolic extracts. Furthermore, it was intended to compare the differentiated activity of each extract against specific bacteria, fungi, and selected human tumor cell lines, using principal component analysis.

## 2. Materials and Methods

### 2.1. Samples

Cultivated* Laurus nobilis *L. samples (leaves) were purchased from Ervital (Castro Daire, Portugal), which produces Mediterranean herbs using organic farming principles and methods. The wild samples (leaves) were collected in Bragança, Portugal, and further lyophilized (FreeZone 4.5, Labconco, KS, USA). Each sample was reduced to a fine dried powder (20 mesh) and stored (7°C) until further use.

### 2.2. Standards and Reagents

Fetal bovine serum (FBS), L-glutamine, Hank's balanced salt solution (HBSS), trypsin-EDTA (ethylenediaminetetraacetic acid), nonessential amino acids solution (2 mM), penicillin/streptomycin solution (100 U/mL and 100 mg/mL, resp.), RPMI-1640, and DMEM media were from HyClone (Logan, UT, USA). Acetic acid, ellipticine, sulforhodamine B (SRB), trypan blue, trichloroacetic acid (TCA), and Tris were from Sigma Chemical Co. (Saint Louis, USA). Mueller-Hinton agar (MH) and malt agar (MA) were obtained from the Institute of Immunology and Virology, Torlak (Belgrade, Serbia). Dimethyl sulfoxide (DMSO) (Merck KGaA, Germany) was used as a solvent. Phosphate buffered saline (PBS) was obtained from Sigma Chemical Co. (St. Louis, USA). Methanol and all other chemicals and solvents were of analytical grade and purchased from common sources. Water was treated in a Milli-Q water purification system (TGI Pure Water Systems, USA).

### 2.3. Extracts Preparation

Methanolic extracts were obtained from cultivated and wild plant material. Each sample (*≈*1 g) was extracted by stirring with 30 mL of methanol, at room temperature, 150 rpm for 1 h. The extract was filtered through Whatman number 4 paper. The residue was then reextracted with additional 30 mL of methanol. The combined extracts were evaporated at 35°C (rotary evaporator Büchi R-210, Flawil, Switzerland) to dryness.

For aqueous extracts, plant material (*≈*1 g) was added to 200 mL of boiling distilled water, left to stand for 5 min out of the heating source, and then filtered under reduced pressure. The obtained extract was frozen and lyophilized.

Methanolic and aqueous extracts were redissolved in water (8 mg/mL) or 5% DMSO (10 mg/mL) for antitumor and antimicrobial activity evaluation, respectively. The final solutions were further diluted to different concentrations for bioactivity evaluation.

### 2.4. Antitumor Activity and Hepatotoxicity

Five human tumor cell lines were tested: MCF7 (breast adenocarcinoma), NCI-H460 (non-small cell lung cancer), HCT15 (colon carcinoma), HeLa (cervical carcinoma), and HepG2 (hepatocellular carcinoma). Cells were routinely maintained as adherent cell cultures in RPMI-1640 medium containing 10% heat-inactivated FBS and 2 mM glutamine (MCF7, NCI-H460, and HCT15) or in DMEM supplemented with 10% FBS, 2 mM glutamine, 100 U/mL penicillin, and 100 mg/mL streptomycin (HeLa and HepG2 cells), at 37°C, in a humidified air incubator containing 5% CO_2_. Each cell line was plated at an appropriate density (7.5 × 10^3^ cells/well for MCF-7, NCI-H460, and HCT15 or 1.0 × 10^4^ cells/well for HeLa and HepG2) in 96-well plates. Sulforhodamine B assay was performed according to a procedure previously described by the authors [27]. Ellipticine was used as positive control.

For hepatotoxicity evaluation, a cell culture (PLP2) was prepared from a freshly harvested porcine liver obtained from a local slaughter house, according to an established procedure [27]. Cell culture was continued with direct monitoring every 2-3 days using a phase contrast microscope. Before confluence was reached, cells were subcultured and plated in 96-well plates at a density of 1.0 × 10^4^cells/well and cultivated in DMEM medium with 10% FBS, 100 U/mL penicillin, and 100 *μ*g/mL streptomycin. Ellipticine was used as positive control. The results were expressed in GI_50_ values (sample concentration that inhibited 50% of the net cell growth).

### 2.5. Antibacterial Activity

The following gram-positive bacteria:* Staphylococcus aureus* (ATCC 6538),* Bacillus cereus* (clinical isolate),* Micrococcus flavus* (ATCC 10240), and* Listeria monocytogenes* (NCTC 7973) and gram-negative bacteria:* Escherichia coli* (ATCC 35210),* Pseudomonas aeruginosa* (ATCC 27853),* Salmonella typhimurium* (ATCC 13311), and* Enterobacter cloacae* (ATCC 35030) were used. The microorganisms were obtained from the Mycological laboratory, Department of Plant Physiology, Institute for Biological Research “Sinisa Stanković” (IBRSS), University of Belgrade, Serbia.

The minimum inhibitory (MIC) and minimum bactericidal (MBC) concentrations were determined by the microdilution method. Briefly, fresh overnight culture of bacteria was adjusted by the spectrophotometer to a concentration of 1 × 10^5^ CFU/mL. The requested CFU/mL corresponded to a bacterial suspension determined in a spectrophotometer at 625 nm (OD625). Dilutions of inocula were cultured on a solid medium to verify the absence of contamination and check the validity of the inoculum. Different solvent dilutions of methanolic extract/fractions were placed in the wells containing 100 *μ*L of tryptic soy broth (TSB) and afterwards 10 *μ*L of inoculum was added. The microplates were incubated for 24 h at 37°C. The MIC of each extract was detected following the addition of 40 *μ*L of iodonitrotetrazolium chloride (INT) (0.2 mg/mL) and incubation at 37°C for 30 min. The lowest concentration that produced a significant inhibition (around 50%) of the growth of the bacteria in comparison with the positive control was identified as the MIC. The minimum inhibitory concentrations (MICs) obtained from the susceptibility testing of various bacteria to tested extract/fraction were determined also by a colorimetric microbial viability assay based on reduction of INT color and compared with positive control for each bacterial strain [28, 29]. MBC was determined by serial subcultivation of 10 *μ*L into microplates containing 100 *μ*L of TSB. The lowest concentration not showing growth after this subculturing was read as the MBC. Standard drugs, namely, streptomycin and ampicillin, were used as positive controls. DMSO (5%) was used as negative control.

### 2.6. Antifungal Activity

For the antifungal bioassays, the following microfungi were used:* Aspergillus fumigatus *(1022),* Aspergillus ochraceus* (ATCC 12066),* Aspergillus versicolor *(ATCC 11730),* Aspergillus niger* (ATCC 6275),* Penicillium funiculosum* (ATCC 36839),* Penicillium ochrochloron *(ATCC 9112),* Penicillium verrucosum *var.* cyclopium* (food isolate), and* Trichoderma viride* (IAM 5061). The organisms were obtained from the Mycological Laboratory, Department of Plant Physiology, IBRSS, Belgrade, Serbia. The micromycetes were maintained on malt agar (MA) and the cultures were stored at 4°C and subcultured once a month [30].

The fungal spores were washed from the surface of agar plates with sterile 0.85% saline containing 0.1% Tween 80 (v/v). The spore suspension was adjusted with sterile saline (*≈*1.0 × 10^3^/*μ*L per well). The inocula were stored at 4°C for further use. Dilutions of the inocula were cultured on solid MA to verify the absence of contamination and to check the validity of the inoculum. MICs determination was performed by a serial dilution technique using 96-well microtitre plates. The extract/fractions were dissolved in 5% solution of DMSO and added to broth malt medium with fungal inoculum. The microplates were incubated for 72 h at 28°C. The lowest concentrations without visible growth (at the binocular microscope) were defined as MIC. The minimum fungicidal concentrations (MFCs) were determined by serial subcultivation of 2 *μ*L in microtitre plates containing 100 *μ*L of malt broth per well and further incubation for 72 h at 28°C. The lowest concentration with no visible growth was defined as the MFC, indicating 99.5% killing of the original inoculum. Bifonazole and ketoconazole were used as positive controls. DMSO (5%) was used as negative control [31].

### 2.7. Statistical Analysis

For wild and cultivated plant material, three samples were used and all the assays were carried out in triplicate. Data were expressed as means ± standard deviations, maintaining the decimal places allowed by the magnitude of standard deviation.

An analysis of variance (ANOVA) with type III sums of squares was performed using the GLM (general linear model) procedure of the SPSS software. The dependent variables were analyzed using 2-way ANOVA with the factors “extract” (E) and “origin” (O). When a statistically significant interaction (E×O) was detected, the two factors were evaluated simultaneously by the estimated marginal means plots for the two levels of each factor. Alternatively, if no statistical significant interaction was verified, means were compared using results obtained for EB and GI that were classified using a simple *t*-test (after checking the equality of variances through Levene's test), since there were fewer than three groups.

Principal components analysis (PCA) was applied as pattern recognition unsupervised classification method. The number of dimensions to keep for data analysis was assessed by the respective eigenvalues (which should be greater than one), by Cronbach's alpha parameter (that must be positive), and also by the total percentage of variance (that should be as high as possible) explained by the number of components selected. The number of plotted dimensions was chosen in order to allow meaningful interpretations.

All statistical tests were performed at a 5% significance level using the SPSS software, version 20.0 (SPSS Inc.).

## 3. Results and Discussion

The interactions among* L. nobilis* origin (cultivated or wild) and extract (methanolic or aqueous) were evaluated to verify if these factors act together to cause changes in phenolic composition and/or biological activities. Results are presented as the mean value of each origin (O), comprising both extracts, as well as the mean value of each extract (E), containing samples from both origins. When the interaction among factors (O×E) was significant (*P* < 0.05), acting itself as a source of variability, the comparison of means could not be performed. In these cases, the presented conclusions were drawn from the estimated marginal means (EMM) plots obtained in each case. When the interaction was not significant, a simple *t*-test (fewer than three groups) was applied to evaluate the equality of means.

### 3.1. Phenolic Compound Groups Present in the Studied* L. nobilis* Extracts

Table 1 summarizes the phenolic compound groups present inmethanolic and aqueous extracts from cultivated and wild* L. nobilis*, as reported in a previous study of our research group [32]. According to those results, cultivated samples showed higher concentrations of flavonols (e.g., quercetin and kaempferol derivatives) and flavones (e.g., luteolin and apigenin derivatives). On the other hand, methanolic extracts had the highest flavan-3-ols contents (e.g., (−)-epicatechin and a procyanidin trimer with an A-type linkage). These differences were maintained after assembling samples according to their origin or extraction type (Table 1), as previously explained. As it can be concluded, cultivated samples had higher contents in total phenolics, especially due to their higher contents in flavonols, since the amounts in flavan-3-ols were similar for both origins. All the quantified phenolic compound groups tended to be higher in methanolic extracts, probably due to the higher temperature used in aqueous extracts [33]. These tendencies were obtained from the EMM plots since the interaction among factors was significant in all cases.

### 3.2. Antitumor Activity of the Studied* L. nobilis* Extracts

The interaction among factors was again significant in all cases, except MCF7 line (Table 2). Considering each factor individually, the origin of laurel had once more higher influence, producing statistically significant differences in all cases except HepG2. Wild laurel presented lower GI_50_ values than cultivated samples but also higher toxicity against nontumor liver primary cells (PLP2; 114 *μ*g/mL). Nevertheless, this sample might have the potential to be used for antitumor proposes, since the GI_50_ values for hepatotoxicity were higher than those obtained for the tumor cell lines (except HepG2). Cultivated samples showed also antitumor activity against NCI-H460, HCT15, and HeLa, since the corresponding GI_50_ values were quite lower than the toxic concentration for PLP2. Differences among aqueous and methanolic extracts were only significant for HCT15 (47 *μ*g/mL in methanolic extracts), HepG2 (144 *μ*g/mL in aqueous extracts), and PLP2 primary liver cells (99 *μ*g/mL in methanolic extracts). The results for the breast carcinoma cell line (MCF7) showed better results when compared to the essential oil of fruits and leaves of wild* L. nobilis* from Lebanon (>400 *μ*g/mL) [13], but lower activity than aqueous extract from wild laurel from Jordan against the same line (88.32% at 50 *μ*g/mL) [8]. Kaileh et al. [15] only reported that the methanolic extract of wild laurel from Palestine showed no cytotoxicity.

### 3.3. Antibacterial Activity of the Studied* L. nobilis* Extracts

Extract type and origin had a significant interaction in the antibacterial activity against all species, except* M. flavus* (Table 3). Cultivated and wild* L. nobilis* were active against all bacteria strains with MICs of 0.04–0.12 mg/mL and 0.046–0.16 mg/mL, respectively. The MBCs were higher than MICs, varying from 0.09 to 0.39 mg/mL for cultivated laurel and from 0.1 to 0.29 mg/mL for wild samples. The effect of laurel origin* per se *was not significant for* S. aureus *(MIC and MBC),* E. coli* (MBC), and* E. cloacae *(MBC). Methanolic extracts were better inhibitors (0.012–0.12 mg/mL) of bacterial growth than the aqueous extracts (0.07–0.20 mg/mL), except for* M. flavus *(*P* = 0.858). In all cases, the inhibitory and bactericidal activities were higher than those obtained for the standard ampicillin. In the case of streptomycin, the inhibitory activity of the extracts was also higher, except for* S. aureus *(cultivated, wild, and aqueous samples),* B. cereus* (wild and aqueous samples), and* L. monocytogenes *(aqueous extract). The results were similar for bactericidal activity, with streptomycin showing higher activity against* S. aureus* (cultivated, wild, and aqueous samples),* B. cereus* (wild and aqueous samples), and* L. monocytogenes *(cultivated and aqueous samples). The bacterial strains more susceptible to cultivated and wild samples were* E. cloacae *and* P. aeruginosa*, respectively. On the other hand, methanolic and aqueous extractswere particularly active against* S. aureus* and* M. flavus*, respectively. Regarding MBC, the results were the same, except for aqueous extract, which proved to have highest bactericidal effect against* E. cloacae *(0.1 mg/mL).

All presented MICs were much better than those obtained by Al-Hussaini and Mahasneh [12] on the ethanolic extracts of* L. nobilis* from Jordan against* S. aureus*,* B. cereus*,* E. coli*,* S. typhimurium,* and* P. aeruginosa*. The same applies to the results obtained by El Malti and Amarouch [23] on the ethanolic extracts of laurel from Morocco against* B. cereus*,* S. aureus*,* L. monocytogenes*,* E. cloacae*,* E. coli,* and* P. aeruginosa* (>2 mg/mL). The inhibitory activity described herein is also higher than that reported using essential oils of laurel from Turkey (MIC values of 5 mg/mL against* E. coli*,* S. aureus*, and* P. aeruginosa*) [9]. However, better results were obtained by Adwan and Mhanna using aqueous extracts of laurel from Palestine against* S. aureus* bacterial strain (<6.1 × 10^−3 ^mg/L), but only when conjugated with enrofloxacin and cephalexin antibiotics [11].

### 3.4. Antifungal Activity of the Studied* L. nobilis* Extracts

The interaction among factors was once more significant in almost all cases, except MIC values for* P. ochrochloron *(*P* = 0.278) and MBC values for* A. niger *(*P* = 0.312) and* P. ochrochloron *(*P* = 0.052) (Table 4). The inhibitory activity on fungal growth was more influenced by extract type, as it can be concluded from the statistically significant differences verified in all cases, except* A. ochraceus *(*P* = 0.077). Methanolic extracts were more active against* A. versicolor*,* Trichoderma viride*,* P. funiculosum, *and* P. ochrochloron*, while aqueous extracts were better in all remaining cases (except, of course,* A. ochraceus*, which gave no differences). Cultivated and wild samples gave MICs varying from 0.01 to 0.17 mg/mL and from 0.02 to 0.3 mg/mL, respectively. In the cases revealing statistically significant differences, cultivated laurel samples gave higher inhibitory activity.

Concerning fungicidal activity, MFCs varied from 0.03 to 0.6 mg/mL and from 0.03 to 0.5 mg/mL for cultivated and wild samples, respectively. Both origins had the same effect on* A. versicolor*,* A. niger,* and* T. viride*. Comparing extract types, MFC varied from 0.016 to 0.7 mg/mL (methanolic extracts) and from 0.046 to 0.3 mg/mL (aqueous extracts). Like it was observed for inhibitory activity, the fungicidal action was more influenced by the type of extract when compared with laurel origin (except* P. funiculosum*).

For both origins and extracts,* A. fumigatus *(only cultivated and aqueous samples in the case of bifonazole),* A. versicolor*,* A. ochraceus*,* T. viride*,* P. funiculosum,* and* P. ochrochloron* showed better activity than bifonazole and ketoconazole.* A. versicolor* and* T. viride* were the most susceptible fungal strains, while* A. niger* and* P. verrucosum *showed the highest resistance. Al-Hussaini and Mahasneh [12] and Simić et al. [21] reported better results on ethanolic extracts and essential oil, respectively, of laurel leaves from Jordan and Serbia and Montenegro against* A. niger*.

### 3.5. Principal Component Analysis (PCA)

**Figure 1:**
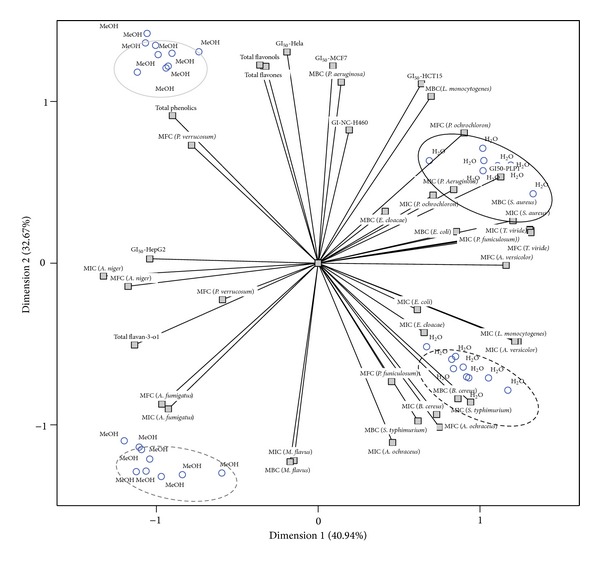
Biplot of objects (extraction solvents) and component loadings (evaluated parameters).

After analysing individually each bioactivity indicator and the contents in phenolic compounds, PCA was applied to understand the true effect of either origin or extract type in a global manner. That is, instead of evaluating individual changes caused in each bioactivity indicator or phenolic compounds group, it was intended to obtain an integrated output dealing with all the effects at once. The plot of component loadings forextract type was designed with the first two dimensions (first: Cronbach's *α*, 0.965; eigenvalue, 17.194; second: Cronbach's *α*, 0.950; eigenvalue, 13.721), which included most variance of data (first: 40.94%; second: 32.67%). The third and fourth dimensions, despite being significant, were not plotted due to the complexity of the corresponding output. The distribution of objects (Figure 1) indicates a clear separation of methanolic and aqueous extracts (black and grey ellipses). Furthermore, objects corresponding to wild and cultivated samples were clearly separated within each type of extract. The assignment of each set of objects to either wild or cultivated samples was done according to the tabled object scores (data not shown).

Group corresponding to cultivated samples extracted with methanol (solid grey line ellipse) was characterized by the high amounts of bioactive compounds, specifically flavonols, flavones, and total phenolics, and its high bioactivity against bacteria (*B. cereus*, MIC and MBC,* S. typhimurium*, MIC and MBC,* E. coli*, MIC,* E. cloacae*, MIC, and* L. monocytogenes*, MIC) and fungi (*A. ochraceus*, MIC and MFC,* A. versicolor*, MIC, and* P. funiculosum*, MFC).

The most distinctive features in cultivated samples extracted with water (solid black line ellipse) were the low content in flavan-3-ols, despite having high antifungal activity against* A. fumigatus *and* A. niger *(MIC and MFC).

A third group (dashed grey line ellipse), corresponding to wild samples extracted with methanol, was characterized as having high antibacterial (*S. aureus*, MIC and MBC,* L. monocytogenes*, MBC, and* P. aeruginosa*, MIC), antifungal (*T. viride*, MIC and MFC,* P. ochrochloron*, MFC, and* P. funiculosum*, MIC), and antitumoral activities (HCT15).

The high bioactivity of wild methanolic extracts might be related to their high content in flavan-3-ols, mainly epicatechin and procyanidin trimer with an A-linkage [32], which were previously reported as having strong antibacterial activity [34, 35]. Curiously, these extracts had an activity opposite to that demonstrated by cultivated samples extracted with water, which might indicate that the* A. fumigatus* and* A. flavus *are poorly susceptible to flavan-3-ols.

Similarly, wild samples extracted with water (dashed black line ellipse) had the reverse behavior in comparison to cultivated samples extracted with methanol. These aqueous extracts were mostly active against* P. verrucosum*, which seemed to have low susceptibility to favonols, flavones, and total phenolics and generally high resistance against the tested extracts (please see Section 3.4).

## 4. Conclusion

The extract type induced the most marked changes in bioactivity of laurel samples. Furthermore, each of the assayed factors (origin and extract type) acts in a differentiated manner; that is, the same evaluated parameter gave sometimes statistically significant differences regarding laurel origin, but no effect at all from extract type or vice versa. From the PCA biplot, it became clear that wild laurel samples were more effective to inhibit tumor cell lines growth, especially HeLa, MCF7, NCI-H460, and HCT15. HepG2, as previously highlighted, had the same reaction to laurel from wild and cultivated origin. It was also observed that methanolic extracts tended to have higher antimicrobial activity, except* A. niger*,* A. fumigatus, *and* P. verrucosum.* The differences in bioactivity might be related to the higher phenolic compounds contents (mainly flavan-3-ols and flavonols) presented by methanolic extracts.

The most interesting finding in this work was the bioactive specificity of each laurel extract, considering its wild or cultivated origin. In fact, from the obtained results it is possible to choose the combination extract type/origin with potentially highest effect against determined bacteria, fungi, or tumor cell line. These findings should, however, be analysed within the geographical area of study, considering eventual specific features of the used samples.

## Figures and Tables

**Table 1:** Phenolic compounds (mg/g) of different extracts of *Laurus nobilis*. The results are presented as mean ± SD.

	Total flavan-3-ols	Total flavones	Total flavonols	Total phenolic
Origin (O)				
Cultivated	56 ± 8	4.4 ± 0.2	26 ± 2	86 ± 11
Wild	60 ± 4	2.6 ± 0.4	7 ± 2	71 ± 6
*P* value (*n* = 18)	**0.025**	**<0.001**	**<0.001**	**<0.001**
Extract (E)				
Methanolic	63.6 ± 0.4	4 ± 1	19 ± 10	86 ± 11
Aqueous	52 ± 5	3 ± 1	15 ± 9	70 ± 5
*P* value (*n* = 18)	**<0.001**	**0.104**	**0.207**	**<0.001**
O×E				
*P* value (*n* = 36)	**<0.001**	**<0.001**	**<0.001**	**<0.001**

The detailed phenolic profile of all laurel samples was previously described by Dias et al. [32].

**Table 2:** Antitumor activity and hepatotoxicity (GI_50_, *μ*g/mL) of different extracts of *Laurus nobilis*. The results are presented as mean ± SD^1^.

	MCF7	NCI-H460	HCT15	HeLa	HepG2	PLP2-hepatotoxicity
Origin (O)						
Cultivated	187 ± 12^a^	83 ± 13	56 ± 1	119 ± 21	185 ± 7	195 ± 85
Wild	88 ± 5^b^	73 ± 19	44 ± 7	69 ± 9	166 ± 59	114 ± 29
*P* value (*n* = 18)	**<0.001**	**0.077**	**<0.001**	**<0.001**	**0.171**	**<0.001**
Extract (E)						
Methanolic	140 ± 50	74 ± 21	47 ± 10	100 ± 41	207 ± 17	99 ± 14
Aqueous	135 ± 53	81 ± 10	53 ± 2	88 ± 11	144 ± 37	210 ± 70
*P* value (*n* = 18)	**0.773**	**0.254**	**0.011**	**0.242**	**<0.001**	**<0.001**
O×E						
*P* value (*n* = 36)	**0.261**	**<0.001**	**<0.001**	**<0.001**	**<0.001**	**<0.001**
Ellipticine	0.91 ± 0.04	1.42 ± 0.01	1.91 ± 0.05	1.14 ± 0.05	3.2 ± 0.5	2.06 ± 0.03

^1^Means within a column with different letters differ significantly (*P* < 0.001).

**Table 3:** Antibacterial activity (MIC and MBC, mg/mL) of different extracts of *Laurus nobilis*. The results are presented as mean ± SD^1^.

	*Staphylococcus aureus *	*Bacillus cereus *	*Micrococcus flavus *	*Listeria monocytogenes *	*Pseudomonas aeruginosa *	*Salmonella typhimurium *	*Escherichia coli *	*Enterobacter cloacae *
MIC								
Origin (O)								
Cultivated	0.06 ± 0.04	0.08 ± 0.04	0.048 ± 0.005	0.1 ± 0.1	0.08 ± 0.04	0.08 ± 0.03	0.12 ± 0.02	0.04 ± 0.01
Wild	0.05 ± 0.05	0.11 ± 0.01	0.101 ± 0.005	0.16 ± 0.05	0.046 ± 0.003	0.11 ± 0.01	0.16 ± 0.05	0.08 ± 0.05
*P* value (*n* = 18)	**0.619**	**0.001**	**<0.001**	**0.030**	**0.001**	**<0.001**	**0.002**	**0.004**
Extract (E)								
Methanolic	0.012 ± 0.005	0.08 ± 0.04	0.08 ± 0.03	0.06 ± 0.04	0.046 ± 0.003	0.07 ± 0.03	0.12 ± 0.02	0.03 ± 0.01
Aqueous	0.10 ± 0.01	0.11 ± 0.01	0.07 ± 0.03	0.20 ± 0.02	0.08 ± 0.03	0.11 ± 0.01	0.16 ± 0.05	0.08 ± 0.05
*P* value (*n* = 18)	**<0.001**	**0.001**	**0.858**	**<0.001**	**0.001**	**<0.001**	**0.009**	**0.002**
O×E								
*P* value (*n* = 36)	**0.002**	**<0.001**	**0.212**	**<0.001**	**<0.001**	**<0.001**	**<0.001**	**<0.001**
Ampicillin	0.25 ± 0.02	0.25 ± 0.03	0.25 ± 0.04	0.37 ± 0.05	0.74 ± 0.05	0.37 ± 0.02	0.25 ± 0.01	0.37 ± 0.04
Streptomycin	0.04 ± 0.01	0.09 ± 0.01	0.17 ± 0.02	0.17 ± 0.01	0.17 ± 0.01	0.17 ± 0.02	0.17 ± 0.02	0.26 ± 0.03

MBC								
Origin (O)								
Cultivated	0.16 ± 0.04	0.15 ± 0.04	0.11 ± 0.01	0.39 ± 0.02	0.18 ± 0.02	0.16 ± 0.05	0.20 ± 0.02	0.09 ± 0.03
Wild	0.1 ± 0.1	0.19 ± 0.02	0.20 ± 0.01	0.29 ± 0.05	0.10 ± 0.01	0.21 ± 0.02	0.2 ± 0.1	0.1 ± 0.1
*P* value (*n* = 18)	**0.262**	**0.001**	**<0.001**	**<0.001**	**<0.001**	**0.002**	**0.764**	**0.111**
Extract (E)								
Methanolic	0.07 ± 0.05	0.15 ± 0.04	0.16 ± 0.05	0.3 ± 0.1	0.14 ± 0.04	0.17 ± 0.05	0.1 ± 0.1	0.08 ± 0.04
Aqueous	0.21 ± 0.02	0.19 ± 0.01	0.15 ± 0.05	0.37 ± 0.03	0.15 ± 0.05	0.21 ± 0.01	0.3 ± 0.1	0.1 ± 0.1
*P* value (*n* = 18)	**<0.001**	**<0.001**	**0.461**	**0.005**	**0.696**	**0.008**	**<0.001**	**0.041**
O×E								
*P* value (*n* = 36)	**<0.001**	**<0.001**	**0.719**	**<0.001**	**<0.001**	**<0.001**	**<0.001**	**<0.001**
Ampicillin	0.37 ± 0.04	0.37 ± 0.05	0.37 ± 0.04	0.49 ± 0.05	1.2 ± 0.1	0.49 ± 0.05	0.49 ± 0.04	0.74 ± 0.05
Streptomycin	0.09 ± 0.01	0.17 ± 0.02	0.34 ± 0.05	0.34 ± 0.04	0.34 ± 0.03	0.34 ± 0.05	0.34 ± 0.04	0.52 ± 0.05

^1^Means within a column with different letters differ significantly (*P* < 0.05).

**Table 4:** Antifungal activity (MIC and MFC, mg/mL) of different extracts of *Laurus nobilis*. The results are presented as mean ± SD^1^.

	*Aspergillus fumigatus *	*Aspergillus versicolor *	*Aspergillus ochraceus *	*Aspergillus niger *	*Trichoderma viride *	*Penicillium funiculosum *	*Penicillium ochrochloron *	*Penicillium verrucosum *
MIC								
Origin (O)								
Cultivated	0.07 ± 0.05	0.01 ± 0.01	0.04 ± 0.01	0.3 ± 0.2	0.02 ± 0.01	0.03 ± 0.01	0.12 ± 0.02	0.17 ± 0.05
Wild	0.2 ± 0.1	0.02 ± 0.01	0.048 ± 0.004	0.3 ± 0.2	0.02 ± 0.01	0.03 ± 0.02	0.11 ± 0.02	0.20 ± 0.02
*P* value (*n* = 18)	**<0.001**	**0.005**	**<0.001**	**0.603**	**0.163**	**0.407**	**0.054**	**0.005**
Extract (E)								
Methanolic	0.2 ± 0.1	0.009 ± 0.003	0.04 ± 0.01	0.47 ± 0.01	0.008 ± 0.005	0.017 ± 0.005	0.10 ± 0.01	0.20 ± 0.02
Aqueous	0.06 ± 0.04	0.024 ± 0.005	0.045 ± 0.002	0.07 ± 0.04	0.029 ± 0.002	0.048 ± 0.002	0.12 ± 0.02	0.17 ± 0.05
*P* value (*n* = 18)	**<0.001**	**<0.001**	**0.077**	**<0.001**	**<0.001**	**<0.001**	**0.008**	**0.007**
O×E								
*P* value (*n* = 36)	**<0.001**	**0.003**	**<0.001**	**<0.001**	**<0.001**	**<0.001**	**0.278**	**<0.001**
Bifonazole	0.15 ± 0.01	0.10 ± 0.01	0.15 ± 0.02	0.15 ± 0.01	0.15 ± 0.01	0.20 ± 0.03	0.20 ± 0.02	0.10 ± 0.01
Ketoconazole	0.20 ± 0.02	0.20 ± 0.03	1.5 ± 0.1	0.20 ± 0.02	1.0 ± 0.1	0.20 ± 0.02	2.5 ± 0.3	0.20 ± 0.04

MFC								
Origin (O)								
Cultivated	0.2 ± 0.1	0.05 ± 0.03	0.08 ± 0.03	0.4 ± 0.4	0.03 ± 0.01	0.10 ± 0.02	0.23 ± 0.02	0.6 ± 0.3
Wild	0.4 ± 0.1	0.04 ± 0.01	0.11 ± 0.01	0.5 ± 0.3	0.03 ± 0.02	0.11 ± 0.02	0.20 ± 0.02	0.40 ± 0.03
*P* value (*n* = 18)	**<0.001**	**0.091**	**<0.001**	**0.196**	**0.500**	**0.027**	**<0.001**	**0.041**
Extract (E)								
Methanolic	0.3 ± 0.1	0.021 ± 0.004	0.08 ± 0.03	0.7 ± 0.3	0.016 ± 0.004	0.10 ± 0.02	0.20 ± 0.03	0.6 ± 0.2
Aqueous	0.2 ± 0.1	0.06 ± 0.02	0.11 ± 0.01	0.2 ± 0.1	0.046 ± 0.002	0.11 ± 0.01	0.23 ± 0.02	0.3 ± 0.1
*P* value (*n* = 18)	**<0.001**	**<0.001**	**<0.001**	**<0.001**	**<0.001**	**0.122**	**<0.001**	**<0.001**
O×E								
*P* value (*n* = 36)	**0.001**	**<0.001**	**<0.001**	**0.312**	**<0.001**	**<0.001**	**0.052**	**<0.001**
Bifonazole	0.20 ± 0.02	0.20 ± 0.03	0.20 ± 0.01	0.20 ± 0.02	0.20 ± 0.04	0.25 ± 0.05	0.25 ± 0.04	0.20 ± 0.03
Ketoconazole	0.50 ± 0.05	0.50 ± 0.04	2.0 ± 0.4	0.50 ± 0.05	1.0 ± 0.1	0.50 ± 0.04	3.5 ± 0.5	0.30 ± 0.05

^1^Means within a column with different letters differ significantly (*P* < 0.05).
